# A unique role for galectin-9 in angiogenesis and inflammatory arthritis

**DOI:** 10.1186/s13075-018-1519-x

**Published:** 2018-02-12

**Authors:** Martin J. O’Brien, Qiang Shu, W.Alexander Stinson, Pei-Suen Tsou, Jeffrey H. Ruth, Takeo Isozaki, Phillip L. Campbell, Ray A. Ohara, Alisa E. Koch, David A. Fox, M. Asif Amin

**Affiliations:** 1 Division of Rheumatology and Clinical Autoimmunity Center of Excellence, Department of Internal Medicine, University of Michigan Medical School, 4368 BSRB, 109 Zina Pitcher Drive, Ann Arbor, MI 48109-2200 USA; 2 Shenzhen Research Institute of Shandong University, Shenzhen, China; 3 Rheumatology Department, Qilu Hospital of Shandong University, Jinan, 250012 China; 4 Department of Veterans Affairs Ann Arbor Healthcare System, Ann Arbor, MI USA

## Abstract

**Background:**

Galectin-9 (Gal-9) is a mammalian lectin secreted by endothelial cells that is highly expressed in rheumatoid arthritis synovial tissues and synovial fluid. Roles have been proposed for galectins in the regulation of inflammation and angiogenesis. Therefore, we examined the contribution of Gal-9 to angiogenesis and inflammation in arthritis.

**Methods:**

To determine the role of Gal-9 in angiogenesis, we performed human dermal microvascular endothelial cell (HMVEC) chemotaxis, Matrigel tube formation, and mouse Matrigel plug angiogenesis assays. We also examined the role of signaling molecules in Gal-9-induced angiogenesis by using signaling inhibitors and small interfering RNA (siRNA). We performed monocyte (MN) migration assays in a modified Boyden chamber and assessed the arthritogenicity of Gal-9 by injecting Gal-9 into mouse knees.

**Results:**

Gal-9 significantly increased HMVEC migration, which was decreased by inhibitors of extracellular signal-regulating kinases 1/2 (Erk1/2), p38, Janus kinase (Jnk), and phosphatidylinositol 3-kinase. Gal-9 HMVEC-induced tube formation was reduced by Erk1/2, p38, and Jnk inhibitors, and this was confirmed by siRNA knockdown. In mouse Matrigel plug assays, plugs containing Gal-9 induced significantly higher angiogenesis, which was attenuated by a Jnk inhibitor. Gal-9 also induced MN migration, and there was a marked increase in MN ingress when C57BL/6 mouse knees were injected with Gal-9 compared with the control, pointing to a proinflammatory role for Gal-9.

**Conclusions:**

Gal-9 mediates angiogenesis, increases MN migration in vitro, and induces acute inflammatory arthritis in mice, suggesting a novel role for Gal-9 in angiogenesis, joint inflammation, and possibly other inflammatory diseases.

## Background

Galectins are a group of mammalian lectins with a high affinity for β-galactosides that share a highly conserved carbohydrate recognition domain (CRD) [1]. Three naturally occurring splice variants of this gene exist, designated Gal-9 short, Gal-9 medium (Gal-9 M), and Gal-9 long, each corresponding to the relative length of the linker peptide connecting the nonhomologous CRDs of Gal-9 [2, 3]. Gal-9 M is a 323-amino acid splice variant of the *LGALS9* gene. Gal-9 causes apoptosis of CD4^+^ T-helper 1 (T_H_1) cells at high concentrations [4, 5], but it activates and expands T_H_1 cell populations at lower concentrations [6]. Gal-9 is highly expressed in rheumatoid arthritis (RA) synovial fluid (SF) and synovial tissue (ST) compared with normal or osteoarthritic ST [7], suggesting a role for Gal-9 in RA.

Angiogenesis is a key aspect of both tumor growth and RA in which the endothelium undergoes morphological changes resulting not only in expansion of the vascular bed but also in increased leukocyte adhesion and infiltration [8, 9]. Angiogenesis is mediated by the mitogen-activated protein kinases (MAPKs), such as extracellular signal-regulating kinases 1/2 (Erk1/2), Janus kinase (Jnk), and p38 [10–14].

In this study, we found that Gal-9 M induces human dermal microvascular endothelial cell (HMVEC) migration and tube formation on Matrigel, as well as in vivo angiogenesis, via the Erk1/2, p38, and Jnk pathways. Gal-9 M induces monocyte (MN) migration and acute inflammation when injected into mouse knees, indicating the role of Gal-9 in angiogenesis and acute inflammation. Gal-9 also induces the phosphorylation of Erk1/2, p38, and Jnk in HMVECs.

## Methods

### HMVEC chemotaxis assays

HMVECs (Lonza, Walkersville, MD, USA) were cultured in endothelial basal media (Lonza). Recombinant human Gal-9 M (Ecalectin; R&D Systems, Madison, WI, USA) was used at various concentrations to perform HMVEC chemotaxis [10–15]. To study the effects of signaling molecules, we performed HMVEC migration assays with Gal-9 in the presence or absence of the following inhibitors: PD98059 (Erk1/2 inhibitor), LY294002 (phosphatidylinositol 3-kinase [PI3K] inhibitor), SB203580 (p38 MAPK inhibitor), and SP600125 (Jnk inhibitor). These inhibitors were purchased from Calbiochem (San Diego, CA, USA). Basic fibroblast growth factor (bFGF) and PBS were used as positive and negative controls, respectively. All the inhibitors were used at 10 μmol/L concentration. To confirm the data obtained with chemical signaling inhibitors, we transfected HMVECs with small interfering RNA siRNA (Santa Cruz Biotechnology, Dallas, TX, USA) of signaling molecules for 48 h using Mirus transfection reagent (Mirus Bio, Madison, WI, USA) and performed HMVEC chemotaxis using Gal-9 M as a stimulus.

### Matrigel in vitro HMVEC tube formation assays

To evaluate the effect of Gal-9 M on capillary morphogenesis, an HMVEC tube formation assay was performed with different concentrations of Gal-9 M using growth factor-reduced (GFR) Matrigel [11, 15]. To determine the effect of signaling molecules on Gal-9 M-induced angiogenesis in vitro, HMVEC tube formation assays were then performed with or without signaling inhibitors. The data obtained with chemical signaling inhibitors was confirmed using siRNAs against various signaling molecules.

### Immunoblotting and cell lysis

HMVECs were stimulated with Gal-9 M (27.9 nmol/L) for various time points. To determine the role of signaling molecules, HMVECs were incubated with chemical signaling inhibitors for 1 h prior to stimulation with Gal-9 M. To ensure equal loading, protein concentrations of samples were determined using the Pierce bicinchoninic acid protein assay kit (Thermo Fisher Scientific, Rockford, IL, USA). Samples were then analyzed by Western blotting using antibodies against phosphorylated Erk1/2, p38, and Jnk (Cell Signaling Technology, Danvers, MA, USA) [12, 16, 17]. Immunoblots were stripped and reprobed with β-actin and antibodies to nonphosphorylated proteins to further ensure equal loading.

### Matrigel plug assay in vivo

Female C57BL/6 mice (aged 6–8 weeks; National Cancer Institute, Bethesda, MD, USA) were injected with GFR Matrigel (500 μl) containing Gal-9 M (L) or PBS (control). Mice were/L) or PBS (control). Mice were euthanized after day 7; the plugs were dissected; and the amount of angiogenesis was determined by hemoglobin measurement [10–13, 15]. Immunofluorescence was performed to evaluate the number of blood vessels present in Gal-9 M- or PBS-injected plug cryosections using von Willebrand factor (vWF) obtained from Dako (Carpinteria, CA, USA) [10–13, 15]. To determine the role of signaling molecules in Gal-9 M-mediated angiogenesis in vivo, the mouse Matrigel plug assay was performed with Gal-9 M in the presence or absence of the signaling inhibitors SB203580 (p38 MAPK inhibitor) and SP600125 (Jnk inhibitor). All experiments performed with animals were done with the approval of the University of Michigan’s University Committee on Use and Care of Animals. The University of Michigan is accredited by the Association for Assessment and Accreditation of Laboratory Animal Care International, and the animal care and use program conforms to the National Institutes of Health standards set forth in the Guide for the Care and Use of Laboratory Animals (revised 2011).

### MN chemotaxis assays

MNs were isolated from normal human blood, and chemotaxis assays were performed with MNs using 48-well modified Boyden chambers (Neuro Probe, Cabin John, MD, USA) as described elsewhere [17–20]. Gal-9 M was used at two different concentrations. PBS and *N*-formylmethionyl-leucyl-phenylalanine (fMLP) served as negative and positive controls, respectively. Each test group was assayed in quadruplicate. Three high-power fields (HPFs) at ×400 magnification were counted in each replicate well by a blinded observer. Written informed consent was obtained from all subjects, and the study was approved by the University of Michigan Institutional Review Board.

### Acute inflammatory arthritis induced by injecting Gal-9 into mouse knees

Female C57BL/6 mice (aged 6–8 weeks) were anesthetized using ketamine (80 mg/kg body weight) on day 0, and knee circumferences were measured. Mice were given intra-articular knee injections of either PBS (20 μl) or Gal-9 M at 139.5 nmol/L or 1.39 μmol/L [18, 20, 21]. After 24 h, mouse knees were measured by an observer blinded to the experimental groups. Mice were then euthanized, and their knees were harvested, stored in optimum cutting temperature medium, and cryosectioned. Immunofluorescence was performed with F4/80 antibody (GeneTex, Irvine, CA, USA) to detect MNs/macrophages and Alexa Fluor 555 goat antirat immunoglobulin G (Thermo Fisher Scientific) was used as a secondary antibody [11, 17, 18].

### Statistical analysis

For statistical analysis, nonparametric Mann-Whitney *U* tests were used to determine statistical significance between various groups for most assays. Where appropriate, Student’s *t* test was used to evaluate significance between groups. Results are expressed as the mean ± SEM. *p* Values less than 0.05 were considered significant.

## Results

### Gal-9 M induces HMVEC migration in vitro

**Fig. 1:**
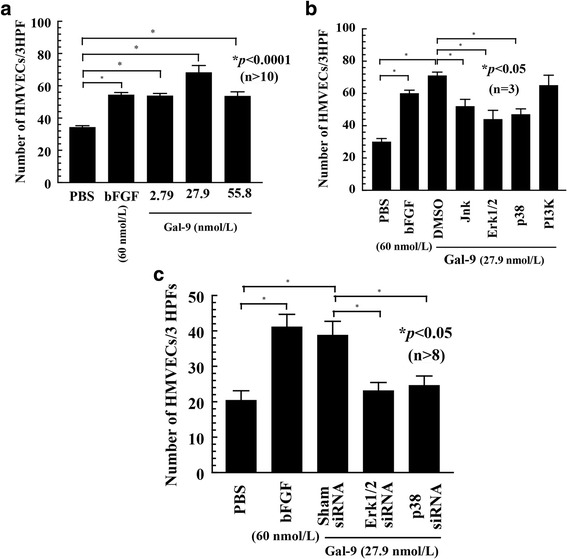
Galectin-9 medium (Gal-9 M) induces human dermal microvascular endothelial cell (HMVEC) migration in vitro. **a** Gal-9 M induced HMVEC migration at concentrations between 2.79 nmol/L and 55.8 nmol/L (*p* < 0.0001). *n* Number of the replicates in each group. **b** HMVEC chemotaxis with various signaling inhibitors. Signaling inhibitors of Janus kinase (Jnk), extracellular signal-regulating kinases 1/2 (Erk1/2), and p38 significantly reduced Gal-9 M-stimulated HMVEC chemotaxis compared with the control group (*p* < 0.05). *n* Number of experiments. **c** A chemotaxis assay was performed with HMVECs transfected with Erk1/2 and p38 small interfering RNA (siRNA) for 48 h. HMVECs transfected with Erk1/2 and p38 siRNAs had significantly less migration in response to Gal-9 M (*p* < 0.05). *n* Number of replicates in each group. *bFGF* Basic fibroblast growth factor, *DMSO* Dimethyl sulfoxide, *HPF* High-power field, *PI3K* Phosphatidylinositol 3-kinase

To determine the role of Gal-9 M in HMVEC chemotaxis, we performed HMVEC migration assays in modified Boyden chambers. We found that Gal-9 M induced HMVEC migration at 2.79 nmol/L, 27.9 nmol/L, and 55.8 nmol/L at levels comparable to the positive control bFGF (*p* < 0.0001). This increase was > 2-fold greater than the negative control PBS (Fig. 1a).

### Gal-9 M induces HMVEC chemotaxis via Erk1/2, Jnk, p38, and PI3K

To assess the signaling pathways necessary for Gal-9 M-stimulated cell migration, HMVEC chemotaxis was performed in the presence or absence of signaling inhibitors. The inhibitors of Erk1/2 (PD98059), Jnk (SP600125), and p38 (SB203580) all significantly inhibited Gal-9 M-mediated HMVEC migration (*p* < 0.05), whereas an inhibitor of PI3K (LY294002) did not (Fig. 1b). To confirm our data, we performed HMVEC chemotaxis with cells transfected with Erk1/2 and p38 siRNA using Gal-9 M as a stimulus. Gal-9 M-mediated HMVEC migration was decreased in cells transfected with Erk1/2 and p38 siRNAs compared with sham (scrambled)-transfected HMVECs (Fig. 1c). Sham (scrambled)-transfected HMVECs were used as positive and negative controls.

### Gal-9 M induces HMVEC tube formation in vitro

**Fig. 2:**
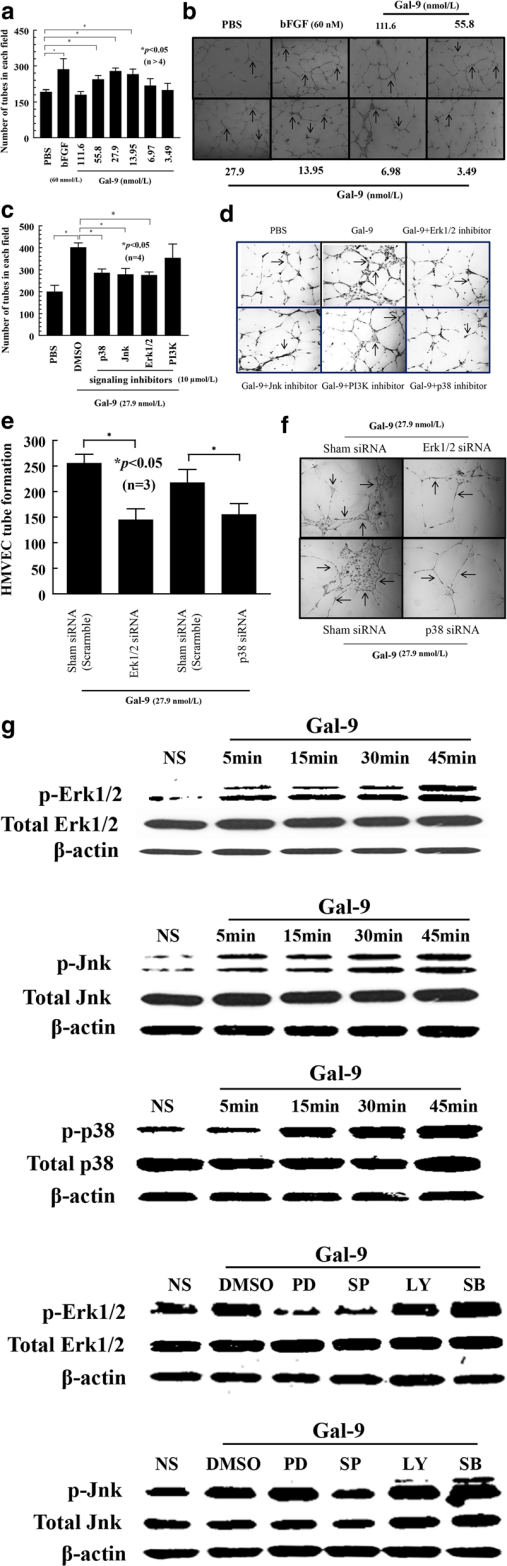
Galectin-9 medium (Gal-9 M) induces human dermal microvascular endothelial cell (HMVEC) tube formation in vitro*.* **a** Gal-9 M induced HMVEC tube formation on growth factor-reduced (GFR) Matrigel that was higher than that induced with PBS (*p* < 0.05). **b** Photomicrographs at ×20 magnification of tube formation assay with PBS, a negative control, and basic fibroblast growth factor, a positive control, and Gal-9 at various concentrations. We did not find a significant increase in HMVEC tube formation in response to Gal-9 M at 111.6, 6.97, and 3.49 nmol/L concentrations. *Arrows* indicate tubes formed in response to each group. **c** Matrigel tube formation assay with Gal-9 using various signaling inhibitors. Inhibitors of p38, Janus kinase (Jnk), and extracellular signal-regulating kinases 1/2 (Erk1/2) significantly reduced Gal-9 M-induced tube formation (*p* < 0.05), whereas the signaling inhibitor of phosphatidylinositol 3-kinase did not. **d** Photographs at ×20 magnification of tube formation assay with various signaling inhibitors. *Arrows* indicate HMVEC tubes formed in response to Gal-9 M with or without signaling inhibitors. **e** and **f** siRNA directed against Erk1/2 and p38 significantly reduced HMVEC tube formation on Matrigel in response to Gal-9 M compared with sham (scrambled)-transfected HMVECs. **g** Western blots with HMVECs stimulated with Gal-9 M. HMVECs were stimulated for 5, 15, 30, and 45 minutes with Gal-9 M (27.9 nmol/L). Shown are one of two representative blots for incremental increases in phosphorylation for Erk1/2, p38, and Jnk proportional to length of time stimulated. Western blots were performed with lysates of HMVECs stimulated with Gal-9 M in the presence or absence of signaling inhibitors. Jnk signaling inhibitor decreased Erk1/2 phosphorylation, suggesting that Jnk is upstream of Erk1/2 in the Gal-9 M-induced signaling pathway. Additionally, Jnk inhibitor did not reduce p38 phosphorylation and vice versa

HMVEC tube formation is a critical indicator of the angiogenic response and reflects capillary morphogenesis. We measured the number of cordlike structures formed after overnight incubation with various concentrations of Gal-9 M from 3.49 nmol/L up to 111.6 nmol/L. We found that Gal-9 M-induced HMVEC migration was significantly higher between 13.95 and 55.8 nmol/L compared with PBS (control) (*p* < 0.05), suggesting a role of Gal-9 M in angiogenesis (Fig. 2a, b). The highest (111.6 nmol/L) and lowest (3.49 and 6.98 nmol/L) concentrations of Gal-9 M did not induce significantly more tube formation, suggesting a precisely regulated effect of Gal-9 on HMVEC tube formation.

### Gal-9 M induces HMVEC tube formation via Erk1/2, p38, and Jnk signaling pathways

HMVEC tube formation assays were performed in the presence or absence of signaling kinase inhibitors. The inhibitors of Erk1/2, Jnk, and p38, but not PI3K, inhibited Gal-9 M-mediated tube formation (*p* < 0.05) (Fig. 2c and d). All inhibitors were used at 10 μmol/L concentrations. HMVECs transfected with Erk1/2 and p38 siRNAs formed significantly fewer tubes with Gal-9 M, confirming the role of Erk1/2 and p38 in Gal-9 M-mediated signaling and endothelial cell (EC) tube formation (Fig. 2e and f).

### Gal-9 M activates phosphorylation of HMVEC Erk1/2, p38, and Jnk

Western blotting experiments were performed to determine the effects of Gal-9 M-induced signaling molecules in HMVECs. We found a time-dependent increase in phosphorylation of Erk1/2, p38, and Jnk kinases in HMVECs stimulated with Gal-9 M. HMVECs were treated with signaling inhibitors for 1 h and stimulated with Gal-9 M for 15 minutes. Western blotting was performed to determine the cross-talk between signaling molecules phosphorylated by Gal-9 M. Jnk phosphorylation was unaffected by inhibitors of p38 or Erk1/2 MAPK, indicating that these molecules were not upstream of Jnk. The phosphorylation of Erk1/2 was decreased by the Jnk inhibitor but not by inhibitors of p38 MAPK, demonstrating that Erk1/2 is downstream of Jnk. These results suggest that Jnk is upstream of Erk1/2 in Gal-9-induced phosphorylation (Fig. 2g).

### Gal-9 M stimulates angiogenesis in vivo

**Fig. 3:**
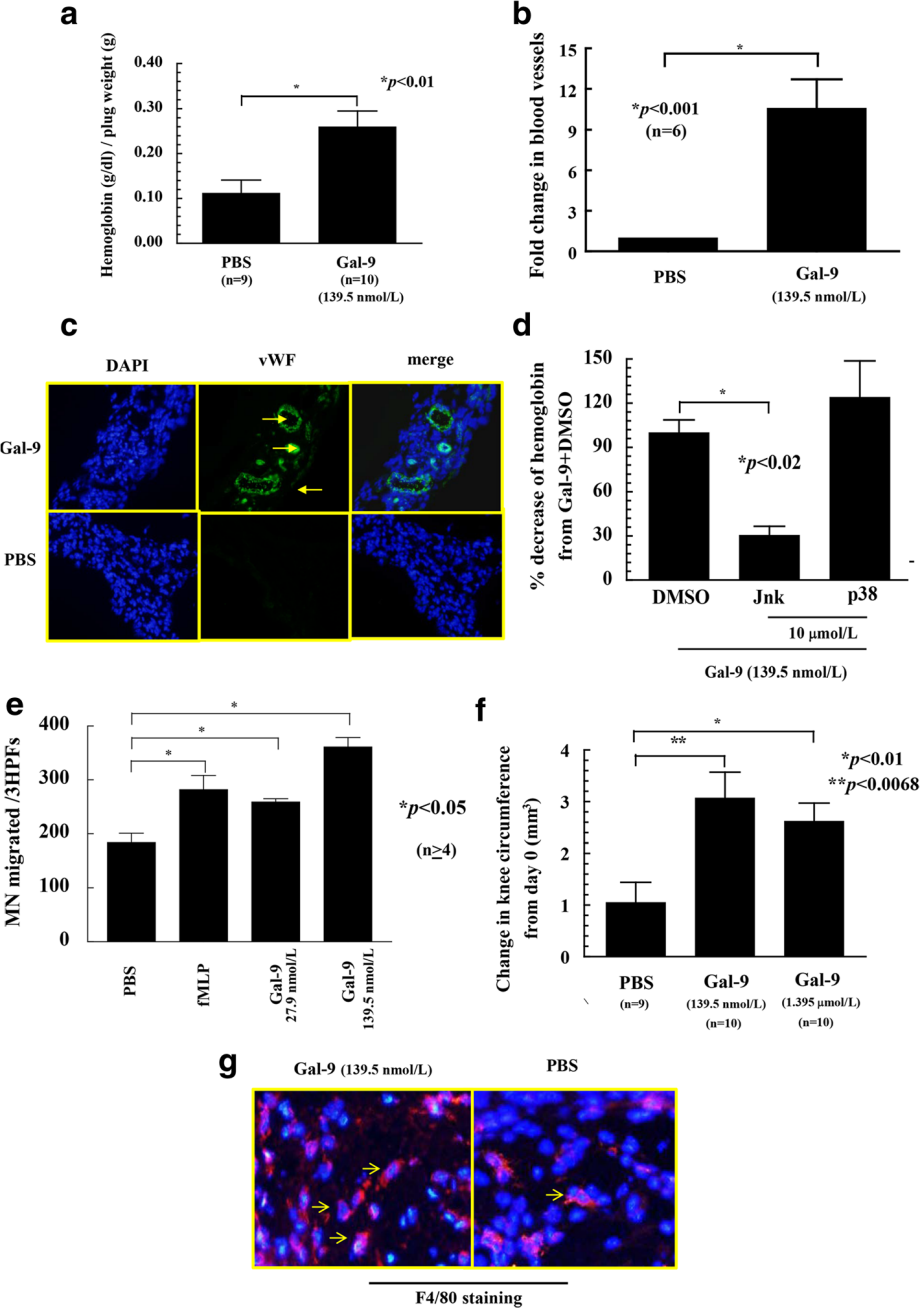
Galectin-9 medium (Gal-9 M) induces angiogenesis and acute inflammation in vivo. **a** Matrigel plug angiogenesis assay with PBS negative control and Gal-9 M (139.5 nmol/L). Matrigel plugs containing Gal-9 M had significantly more hemoglobin than the PBS control group (*p* < 0.01). *n* Number of mice per group. **b** and **c** Cryosection of Matrigel plugs stained for von Willebrand factor (vWF) displayed a significant increase in blood vessel formation in response to Gal-9 M compared with PBS, a negative control (*p* < 0.001). **d** Matrigel plug assay with Janus kinase (Jnk) and p38 signaling inhibitors. Jnk inhibitor significantly reduced Gal-9 M-stimulated angiogenesis in the Matrigel plugs, whereas p38 inhibitor did not. *n* Number of mice per group (*p* < 0.02). **e** Gal-9 M induces in vitro monocyte (MN) chemotaxis. MN chemotaxis assays were performed using a modified Boyden chamber. MN migration was determined in response to Gal-9 M. PBS and *N*-formylmethionyl-leucyl-phenylalanine (fMLP) were used as negative and positive controls, respectively. Three high-power fields (HPFs) were counted per well, and the assay was performed in quadruplicate. *n* Number of replicates. Means are presented with SEM, and *p* values < 0.05 were considered significant. **f** Acute inflammatory arthritis in mouse knees injected with Gal-9 M. Results were measured as knee circumference from the day of injection (day 0). Gal-9 M at concentrations of 139.5 nmol/L and 1.39 μmol/L in 20 μl of PBS induced significantly higher inflammation. At 139.5 nmol/L, knee circumference was increased by ~ 3-fold (*p* < 0.0068), whereas at 1.39 μmol/L, the increase in knee circumference was 2-fold (*p* < 0.01). **g** F4/80 staining of mouse knee cryosections injected with Gal-9 M and PBS. Knees were snap-frozen and cryosectioned, then stained with 4′,6-diamidino-2-phenylindole (DAPI; *blue*) and F4/80 (*red*). Gal-9 M-injected knees had increased MN/macrophage staining compared with those injected with PBS

After finding that Gal-9 M induces HMVEC migration and tube formation in vitro, we examined whether Gal-9 M induces angiogenesis in vivo by performing Matrigel plug assays. Hemoglobin levels, an indirect measure of angiogenesis, were ~ 2-fold higher in plugs containing Gal-9 M compared with PBS control (*p* < 0.01) (Fig. 3a). Some of the plugs were cryosectioned and stained for vWF using rabbit antimouse vWF antibody (Dako). We found a significant increase in blood vessels in the Matrigel plugs injected with Gal-9 M compared with PBS-injected plugs (*p* < 0.001) (Fig. 3b and c).

### Jnk signaling pathway contributes to Gal-9 M-mediated angiogenesis in vivo

To determine the effect of signaling inhibitors in Gal-9 M-mediated angiogenesis in vivo, Matrigel plug assays were performed using chemical signaling inhibitors at a 10 μmol/L concentration. Gal-9 M-induced angiogenesis was significantly reduced in Matrigel plugs containing an inhibitor of Jnk (SP600125) (*p* < 0.02), whereas the signaling inhibitor of p38 had no effect (Fig. 3d). We found a significant decrease in Gal-9 M-mediated HMVEC migration and tube formation in the presence of p38 inhibitor in vitro; yet, the p38 inhibitor was unable to inhibit Gal-9 M-induced angiogenesis in vivo. This suggests that the concentration of the inhibitor might not be maintained locally in vivo and/or that other signals available exclusively in vivo might be able to overcome the effect of the inhibitor.

### Gal-9 M induces MN chemotaxis in vitro

We used two concentrations of Gal-9 M (27.9 nmol/L and 139.5 nmol/L) to examine its role in MN migration. At both concentrations, Gal-9 M-induced MN migration was significantly higher than with PBS control (*p* < 0.05), suggesting that Gal-9 M is a potent human MN chemoattractant (Fig. 3e). We did not find a significant difference in MN migration in response to two different concentrations of Gal-9 M.

### Gal-9 M causes acute inflammation in mouse knees

Gal-9 M was injected into mouse knees to determine whether Gal-9 M had proinflammatory effects when injected directly into mouse knees. Knees injected with 139.5 nmol/L of Gal-9 M exhibited a 3-fold increase in knee circumference (*p* < 0.0068) compared with PBS. Comparatively, knees injected with 1.39 μmol/L showed an ~ 2-fold increase in knee circumference (*p* < 0.01) (Fig. 3f). F4/80-positive MNs/macrophages were markedly elevated in Gal-9 M-injected knees compared with those injected with PBS (Fig. 3g).

## Discussion

Gal-9 is currently known as a versatile immunomodulator that affects a host of cell types, including vascular ECs and leukocytes. The complex and even conflicting functions of Gal-9 can be attributed to the location of its binding to glycosylated proteins, disparate properties of its isoforms, the unique N- and C-CRD structure, and different receptors, such as T-cell immunoglobulin mucin 3 (TIM-3) on immunocytes [1]. Gal-9 also binds to protein disulfide isomerase on T cells and thereby influences T-cell migration by increasing the reduction of disulfide bonds on integrins [22].

The exact contribution of Gal-9 in EC biology and angiogenesis remains elusive. Discrepancies regarding the role of Gal-9 in angiogenesis may be based on the existence of alternative splice variants, the concentrations of Gal-9 employed in various assays, and the types of ECs used [3]. Heusschen et al. found that Gal-9 M enhanced sprout formation and migration of primary ECs, two aspects of angiogenesis, using human umbilical vein endothelial cells. However, the same research group observed a contradictory effect of Gal-9 M on human microvascular endothelial cell-1 (HMEC-1), a human EC line, whereby Gal-9 M decreased proliferation and migration of HMEC-1 cells [3]. Our data support the notion that Gal-9 M contributes to migration and tube formation in primary human ECs. Our data are also in agreement with previous reports that higher concentrations of Gal-9 might be involved in apoptosis of ECs, because we found that Gal-9 triggered HMVEC apoptosis on GFR Matrigel at relatively high concentrations (data not shown), whereas lower concentrations of Gal-9 M induced HMVEC tube formation on GFR Matrigel (Fig. 2).

Gal-9 M induced phosphorylation of Jnk, p38, and Erk1/2 MAPK in HMVECs in a time-dependent manner. In terms of Gal-9 M signaling in HMVECs, we found cross-talk between Jnk and Erk1/2 MAPK, because an inhibitor of Jnk decreased Jnk and Erk1/2 phosphorylation, suggesting that Jnk is upstream of Erk1/2.

Heusschen et al. found that Gal-9 M induced angiogenesis in the chick chorioallantoic membrane assay [3]. Our data are in agreement with those of Heusschen et al. because we found a significant increase in blood vessel formation in response to Gal-9 M in the Matrigel plug angiogenesis assay, another murine model of angiogenesis. The role of signaling molecules such as Erk1/2, Jnk, and p38 in angiogenesis is well known [11, 15]. Erk1/2, Jnk, and p38 mediate Gal-9 M-induced HMVEC migration and Matrigel tube formation. After finding a role for these signaling molecules in Gal-9 M-induced angiogenesis in vitro, we tested their contribution to Gal-9 M-induced angiogenesis in vivo. Jnk contributed to Gal-9 M-induced angiogenesis in the Matrigel plug assay. In contrast, p38 MAPK did not appear to be required in Gal-9-induced angiogenesis in vivo.

The effects of galectins are complex and vary depending upon the route of administration, concentration, intracellular or extracellular localization, and the type of inflammatory model used. There are some controversies regarding the functions of Gal-9 in inflammatory models of arthritis. Anderson et al. found that Gal-9 and its receptor TIM-3 contribute to inflammation by increasing proinflammatory cytokines such as tumor necrosis factor-α (TNF-α) expression from MNs and dendritic cells, whereas Arikawa et al. found that Gal-9 suppresses murine arthritis and TNF-α secretion [23, 24]. Gal-9 is a chemoattractant for neutrophils and eosinophils, but scant evidence exists to support a role for Gal-9 in acute models of inflammation that involve MN recruitment [25, 26]. Our data support the notion that Gal-9 contributes to leukocyte migration and induces MN migration in vitro and in vivo, indicating involvement of Gal-9 in acute inflammation [25, 26].

Some studies suggest that Gal-9 functions as a negative regulator in immune complex-associated arthritis models [7, 23, 27]. In these studies, the effect of Gal-9 M was examined in chronic inflammatory arthritis development by injecting Gal-9 systemically in models that are T-cell-dependent. In contrast to researchers in the above-described studies, Iqbal et al. did not find a decrease in inflammation during the first 24 h when Gal-9 M was injected systemically in the mouse carrageenan paw edema model, an acute model of inflammation [28]. Our data are in agreement with those of Iqbal et al. in indicating that Gal-9 M induces significantly higher inflammation and MN migration when injected directly into mouse knees. Notably, intra-articular but not systemic injection of Gal-9 M would be expected to generate a chemotactic gradient from the circulation into the joint. This suggests that the effect of Gal-9 M depends upon the route of administration and the type of murine inflammatory model used.

## Conclusions

This study suggests that Gal-9 induces angiogenesis and that Jnk, Erk1/2, and p38 play an important role in Gal-9 M-mediated angiogenesis. The involvement of Gal-9 M in MN recruitment renders it a novel therapeutic target in angiogenesis and acute inflammatory conditions.

## Abbreviations

bFGF: Basic fibroblast growth factor
CRD: Carbohydrate recognition domain
DAPI: 4′,6-diamidino-2-phenylindole
DMSO: Dimethyl sulfoxide
EC: Endothelial cell
Erk1/2: Extracellular signal-regulating kinases 1/2
fMLP: *N*-formylmethionyl-leucyl-phenylalanine
Gal-9 M: Galectin-9 medium
GFR: Growth factor-reduced
HMEC-1: Human microvascular endothelial cell-1
HMVEC: Human dermal microvascular endothelial cell
HPF: High-power field
Jnk: Janus kinase
MAPK: Mitogen-activated protein kinase
MN: Monocyte
PI3K: Phosphatidylinositol 3-kinase
RA: Rheumatoid arthritis
SF: Synovial fluid
siRNA: Small interfering RNA
ST: Synovial tissue
T_H_1: T-helper 1 cell
TIM-3: T-cell immunoglobulin mucin 3
TNF-α: Tumor necrosis factor-α
vWF: von Willebrand factor

## References

[1] Barondes SH, Cooper DN, Gitt MA, Leffler H (1994). Galectins: structure and function of a large family of animal lectins. J Biol Chem.

[2] Heusschen R, Griffioen AW, Thijssen VL (2013). Galectin-9 in tumor biology: a jack of multiple trades. Biochim Biophys Acta.

[3] Heusschen R, Schulkens IA, van Beijnum J, Griffioen AW, Thijssen VL (2014). Endothelial LGALS9 splice variant expression in endothelial cell biology and angiogenesis. Biochim Biophys Acta.

[4] Zhu C, Anderson AC, Schubart A, Xiong H, Imitola J, Khoury SJ (2005). The Tim-3 ligand galectin-9 negatively regulates T helper type 1 immunity. Nat Immunol.

[5] Oomizu S, Arikawa T, Niki T, Kadowaki T, Ueno M, Nishi N (2012). Cell surface galectin-9 expressing Th cells regulate Th17 and Foxp3^+^ Treg development by galectin-9 secretion. PLoS One.

[6] Gooden MJ, Wiersma VR, Samplonius DF, Gerssen J, van Ginkel RJ, Nijman HW (2013). Galectin-9 activates and expands human T-helper 1 cells. PLoS One.

[7] Seki M, Sakata KM, Oomizu S, Arikawa T, Sakata A, Ueno M (2007). Beneficial effect of galectin 9 on rheumatoid arthritis by induction of apoptosis of synovial fibroblasts. Arthritis Rheum.

[8] Szekanecz Z, Besenyei T, Szentpetery A, Koch AE (2010). Angiogenesis and vasculogenesis in rheumatoid arthritis. Curr Opin Rheumatol.

[9] Szekanecz Z, Koch AE (2005). Endothelial cells in inflammation and angiogenesis. Curr Drug Targets Inflamm Allergy.

[10] Amin MA, Rabquer BJ, Mansfield PJ, Ruth JH, Marotte H, Haas CS (2010). Interleukin 18 induces angiogenesis in vitro and in vivo via Src and Jnk kinases. Ann Rheum Dis.

[11] Amin MA, Volpert OV, Woods JM, Kumar P, Harlow LA, Koch AE (2003). Migration inhibitory factor mediates angiogenesis via mitogen-activated protein kinase and phosphatidylinositol kinase. Circ Res.

[12] Kumar P, Amin MA, Harlow LA, Polverini PJ, Koch AE (2003). Src and phosphatidylinositol 3-kinase mediate soluble E-selectin-induced angiogenesis. Blood.

[13] Park CC, Morel JC, Amin MA, Connors MA, Harlow LA, Koch AE (2001). Evidence of IL-18 as a novel angiogenic mediator. J Immunol.

[14] Hu X, Mendoza FJ, Sun J, Banerji V, Johnston JB, Gibson SB (2008). Lysophosphatidic acid (LPA) induces the expression of VEGF leading to protection against apoptosis in B-cell derived malignancies. Cell Signal.

[15] Tsou PS, Ruth JH, Campbell PL, Isozaki T, Lee S, Marotte H (2013). A novel role for inducible Fut2 in angiogenesis. Angiogenesis.

[16] Amin MA, Mansfield PJ, Pakozdi A, Campbell PL, Ahmed S, Martinez RJ (2007). Interleukin-18 induces angiogenic factors in rheumatoid arthritis synovial tissue fibroblasts via distinct signaling pathways. Arthritis Rheum.

[17] Amin MA, Ruth JH, Haas CS, Pakozdi A, Mansfield PJ, Haghshenas J (2008). H-2g, a glucose analog of blood group H antigen, mediates mononuclear cell recruitment via Src and phosphatidylinositol 3-kinase pathways. Arthritis Rheum.

[18] Yoshida K, Korchynskyi O, Tak PP, Isozaki T, Ruth JH, Campbell PL (2014). Citrullination of epithelial neutrophil-activating peptide 78/CXCL5 results in conversion from a non-monocyte-recruiting chemokine to a monocyte-recruiting chemokine. Arthritis Rheum.

[19] Kumar P, Hosaka S, Koch AE (2001). Soluble E-selectin induces monocyte chemotaxis through Src family tyrosine kinases. J Biol Chem.

[20] Ruth JH, Park CC, Amin MA, Lesch C, Marotte H, Shahrara S (2010). Interleukin-18 as an in vivo mediator of monocyte recruitment in rodent models of rheumatoid arthritis. Arthritis Res Ther.

[21] Mor-Vaknin N, Saha A, Legendre M, Carmona-Rivera C, Amin MA, Rabquer BJ (2017). DEK-targeting DNA aptamers as therapeutics for inflammatory arthritis. Nat Commun.

[22] Bi S, Hong PW, Lee B, Baum LG (2011). Galectin-9 binding to cell surface protein disulfide isomerase regulates the redox environment to enhance T-cell migration and HIV entry. Proc Natl Acad Sci U S A.

[23] Arikawa T, Watanabe K, Seki M, Matsukawa A, Oomizu S, Sakata KM (2009). Galectin-9 ameliorates immune complex-induced arthritis by regulating FcγR expression on macrophages. Clin Immunol.

[24] Anderson AC, Anderson DE, Bregoli L, Hastings WD, Kassam N, Lei C (2007). Promotion of tissue inflammation by the immune receptor Tim-3 expressed on innate immune cells. Science.

[25] Tsuboi Y, Abe H, Nakagawa R, Oomizu S, Watanabe K, Nishi N (2007). Galectin-9 protects mice from the Shwartzman reaction by attracting prostaglandin E_2_-producing polymorphonuclear leukocytes. Clin Immunol.

[26] Matsumoto R, Matsumoto H, Seki M, Hata M, Asano Y, Kanegasaki S (1998). Human ecalectin, a variant of human galectin-9, is a novel eosinophil chemoattractant produced by T lymphocytes. J Biol Chem.

[27] Seki M, Oomizu S, Sakata KM, Sakata A, Arikawa T, Watanabe K (2008). Galectin-9 suppresses the generation of Th17, promotes the induction of regulatory T cells, and regulates experimental autoimmune arthritis. Clin Immunol.

[28] Iqbal AJ, Sampaio AL, Maione F, Greco KV, Niki T, Hirashima M (2011). Endogenous galectin-1 and acute inflammation: emerging notion of a galectin-9 pro-resolving effect. Am J Pathol.

